# CSF HIV RNA Escape in Opsoclonus-Myoclonus-Ataxia Syndrome: Case Report and Review of the Literature

**DOI:** 10.3389/fneur.2020.585527

**Published:** 2020-11-23

**Authors:** Pierre Cabaraux, Arthur Poncelet, Jérome Honnorat, Remy Demeester, Soraya Cherifi, Mario Manto

**Affiliations:** ^1^Unité des Ataxies Cérébelleuses, Service de Neurologie, Centre Hospitalier Universitaire (CHU)-Charleroi, Charleroi, Belgium; ^2^Service de Médecine Interne, Centre Hospitalier Universitaire (CHU)-Charleroi, Charleroi, Belgium; ^3^Centre National de Référence pour les Syndromes Neurologiques Paranéoplasiques, Hospices Civils de Lyon, Synatac Team, NeuroMyoGene Institute, INSERM U1217/CNRS UMR5310, University Claude Bernard Lyon 1, Université de Lyon, Lyon, France

**Keywords:** HIV, CSF escape, opsoclonus myoclonus ataxia syndrome, cerebellum, sinus thrombosis

## Abstract

**Background:** Human immunodeficiency viruses (HIV) infection is associated with a broad range of neurological manifestations, including opsoclonus-myoclonus ataxia syndrome (OMAS) occurring in primary infection, immune reconstitution syndrome or in case of opportunistic co-infection.

**Case:** We report the exceptional case of a 43-year-old female under HIV treatment for 10 years who presented initially with suspected epileptic seizure. Although the clinical picture slightly improved under anti-epileptic treatment, it was rapidly attributed to OMAS. The patient exhibited marked opsoclonus, mild dysarthria, upper limbs intermittent myoclonus, ataxia in 4 limbs, truncal ataxia, and a severe gait ataxia (SARA score: 34). The diagnostic work-up showed radiological and biological signs of central nervous system (CNS) inflammation and cerebral venous sinus thromboses. The HIV viral load was higher in cerebrospinal fluid (CSF) than in the blood (4,560 copies/ml vs. 76 copies/ml). She was treated for 5 days with pulsed corticotherapy. Dolutegravir and anticoagulation administration were initiated. Follow-ups at 2 and 4 months showed a dramatic improvement of clinical neurologic status (SARA score at 4 months: 1), reduction of CNS inflammation and revealed undetectable CSF and serum viral loads.

**Conclusion:** This case underlines the importance of the evaluation of the CSF viral load in HIV patients developing OMAS and suggests CSF HIV RNA escape as a novel cause for OMAS.

## Introduction

Growing evidence suggests that CNS is a reservoir for HIV. HIV infection has been associated with a broad range of neurological manifestations so far (1). CSF HIV RNA escape is defined as the presence of HIV RNA in greater amount in CSF than in plasma and is known to trigger neurological manifestations (2, 3) even when the infection appears controlled (2).

Opsoclonus-Myoclonus-Ataxia syndrome (OMAS) is a rare auto-immune cerebellar disorder. The etiology is paraneoplastic, para-infectious, toxic-metabolic, or idiopathic (4–6). OMAS has been reported in association with HIV under three conditions: when the infection is newly diagnosed, during immune reconstitution or when another infection occurs (5). We report a case of CSF HIV RNA escape manifesting with OMAS and sinus thrombosis.

## Case Report

A 43 years old female was admitted for suspected epileptic seizure on 13th of December 2019. She had a 10 years history of HIV with three episodes of seizures due to neuro-toxoplasmosis. She was allergic to lamotrigine and cotrimoxazole. Viral load was undetectable in serum on 26th of November 2019 and CD4+ and CD4/CD8 ratio were, respectively at 670 cells/mm^3^ and 0.5. Her treatment consisted in lamivudine, abacavir, darunavir, ritonavir, and clindamycine. She willingly stopped topiramate due to a desire of pregnancy 2 weeks before admission. Her HIV medication was shifted from dolutegravir, darunavir and cobicistat to her actual medication 6 months earlier.

On admission, although the clinical picture slightly improved under anti-epileptic treatment, it was rapidly attributed to OMAS, presenting with marked opsoclonus, mild dysarthria, upper limbs intermittent myoclonus, ataxia in four limbs and truncal ataxia. Gait was severely ataxic. Scale for assessment and rating of ataxia (SARA) score (7) was evaluated at 34. Her husband reported inappropriate attitude with childlike behavior starting 6 months earlier.

**Figure 1:**
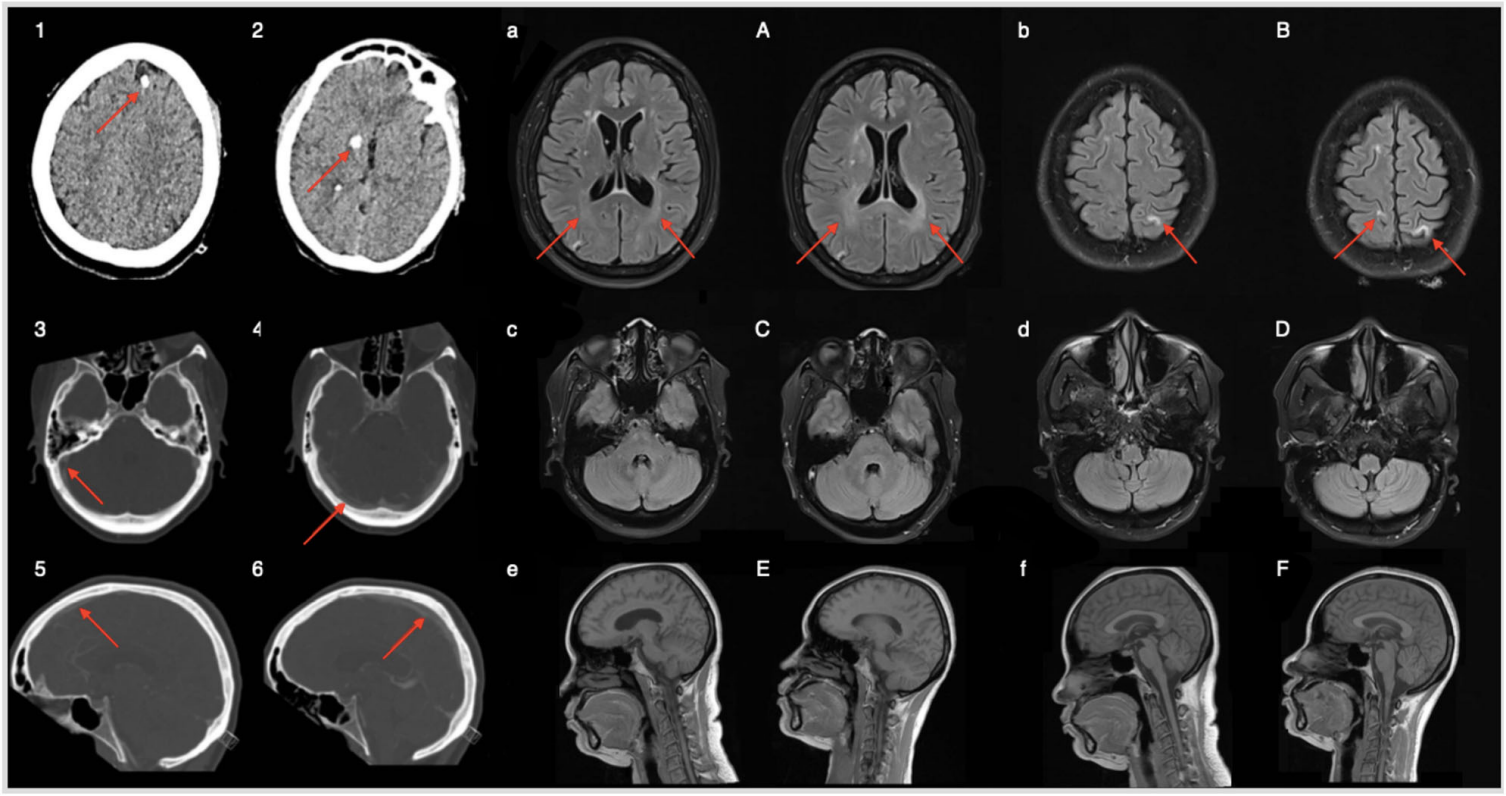
Brain CT on admission **(**panels **1–2)** shows sequellar macro calcification of former neurotoxoplasmosis episodes (red arrows). Panels **(3–6)** refer to brain CT with angiography (venous phase); thrombosis of superior sagittal and right transverse sinus. Brain MRI realized 6 months before admission **(a–f)** and on admission **(A–F)**. White matter hyperintensities in T2 FLAIR account for CSF HIV escape related progressive neuroinflammation. Absence of lesions in the posterior fossa.

Blood samples didn't revealed any significant abnormality and brain CT was unremarkable. Serological work-up was negative for auto-immune diseases or acute infection. Anti-Ri or anti-GlyR antibodies were absent in the serum. Thyroid function, vitamins and iron profiles were normal. CD4+ count was 512 cells/mm^3^, CD4/CD8 ratio was 0.5 and serum HIV viral load was 76 copies RNA/mL. A lumbar puncture (LP) showed a CSF HIV viral load of 4,560 copies/mL, 95/mm^3^ leucocytes (95% lymphocytes), and 113.8 mg/dL proteins. Oligoclonals bands restricted to the CSF were found. CSF autoimmune and infectious encephalitis panel were unremarkable. Full body PET-CT (positron emission tomography with scanner) didn't reveal any hypermetabolism. Brain magnetic resonance imaging (MRI) showed a venous sinus thrombosis (superior sagittal and lateral; confirmed by (computed tomography) CT angiogram in venous phase) as well as periventricular and intra-parenchymatous white matter hyper-intensities in T2-FLAIR as compared to brain MRI realized on June 2019 (Figure 1).

She was treated by a 5 days pulsed corticotherapy and gabapentin. Dolutegravir was added to her antiretroviral therapy. Dabigatran was prescribed for venous thrombosis. Opsoclonus, myoclonus and truncal ataxia rapidly improved within the first 2 weeks of treatment.

**Figure 2:**
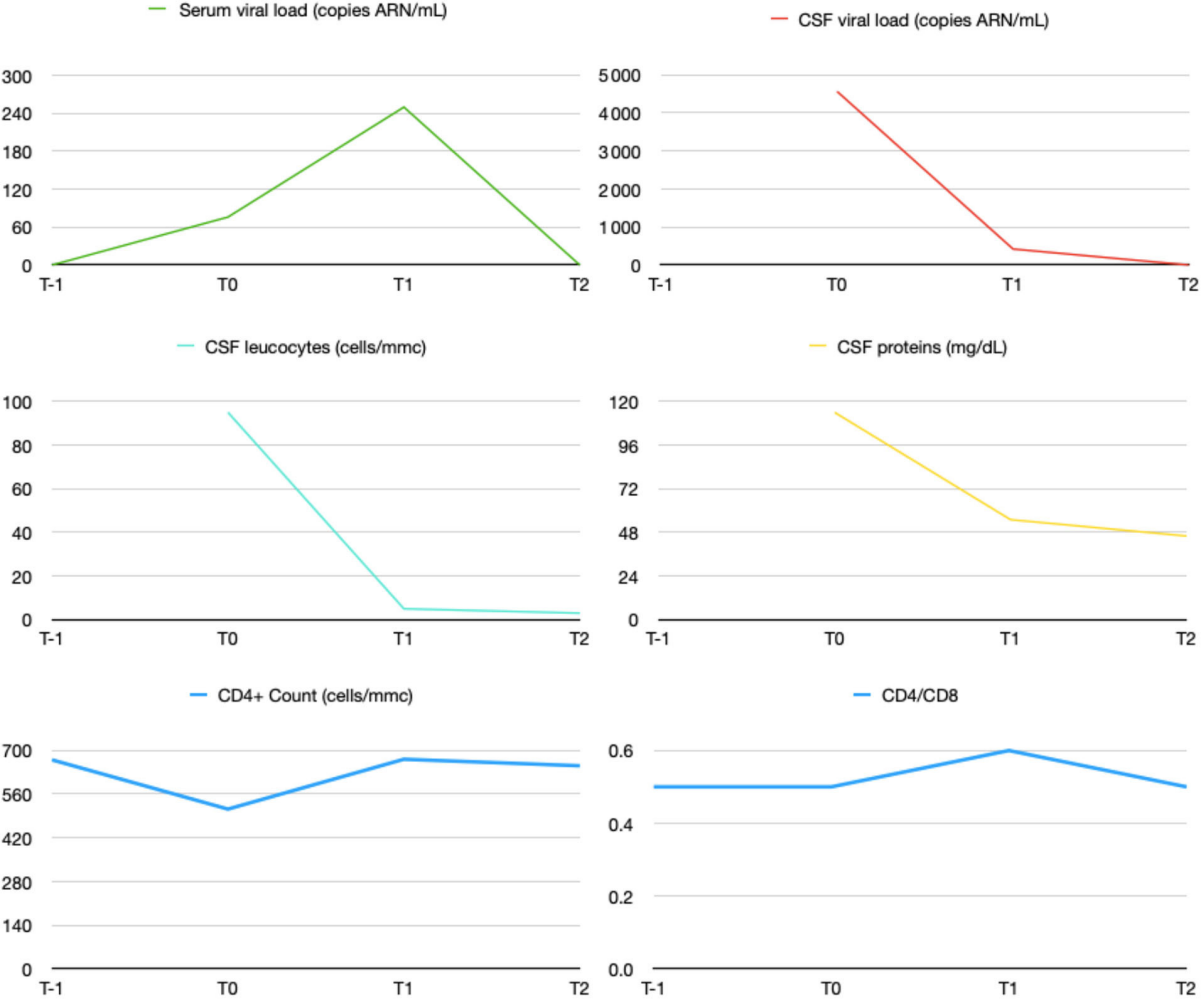
Evolution of CSF and serum HIV viral load and CNS inflammation markers under treatment. Patient was admitted in our hospital on 13th of December 2019. ≪ T-1 ≫ accounts for 26th of November 2019, ≪ T0 ≫ for 13th of December 2019, ≪ T1 ≫ for 10th of February 2020 and ≪ T2 ≫ for 14th of April 2020.

Serum and CSF HIV viral load measurements were repeated on the 10th of February 2020 and 14th of April 2020 as well as CD4+ and CD8+ count. Decrease in both CSF and serum viral load as well as CSF inflammatory markers were found (Figure 2). On April, she had recovered full functional autonomy and was able to walk in tandem gait for 10 steps without mis-step. Her husband noted a marked improvement in her behavior. SARA scale score was evaluated at one.

## Discussion

OMAS is a rare auto-immune mediated cerebellar disorder. The etiology is paraneoplastic, para-infectious, toxic-metabolic, or idiopathic (4–6). Given the relative beneficial effect of immunosuppressive therapy in para-infectious OMAS and presence of antibodies in paraneoplastic-related OMAS, pathogenesis is thought to be autoimmune (4, 8). Transient neuronal dysfunction due to antibodies impairing inhibition between cerebellar cortex upon cerebellar nuclei has been suggested by electrophysiological and imaging studies (4, 6, 8).

In our case, the diagnosis of OMAS was based on the concomitant presence of opsoclonus, myoclonus, ataxia, and behavioral changes [(4); Table 1]. However, it was unclear whether the 6 months childlike behavior history reported by the patient's husband was due to OMAS or CSF HIV RNA escape condition, as CSF HIV infection can induce a wide spectrum of neurological manifestations including behavioral changes (3). Absence of hypermetabolism on PET-CT and onconeuronal antibodies reasonably dismissed oncologic etiology and high CSF compared to serum HIV viral load suggested a causative link between uncontrolled infection and OMAS as previously observed (9). We found no association reported between sinus thrombosis and OMAS. HIV-related OMAS has been observed so far in primo-infection, immune reconstitution or when another infection is associated (5). Eighteen case reports including ours have been reported [(5, 9); Supplementary Files]. Only one report has shown a high CSF compared to serum viral load despite HIV infection control under antiretroviral therapy (ART) in the serum (9). Interestingly, since no antibody has never been found in cases of OMAS related to HIV, the mechanism by which the syndrome occurred remains unclear. We suggest that neuro-inflammation due to the virus in the CNS may play a role that needs to be precise.

**Table 1:** From (4). Proposed diagnostic criteria for opsoclonus myoclonus syndrome (OMS).

**At least 3 of 4 supportive findings**
Opsoclonus
Myoclonus and/or ataxia
Behavioral change and/or sleep disturbance
Tumorous conditions and/or presence of anti-neuronal antibodies

This phenomenon, so called CSF HIV RNA escape is defined as the presence of HIV RNA in greater amount in CSF than in plasma (10). It is estimated to occur in 4–20% of ART experienced HIV-infected adults worldwide (11). Patients are either symptomatic or asymptomatic with many clinical manifestations ranging from progressive mild neurocognitive impairment to seizures or acute alteration of consciousness (3).

Pathogenesis of CSF escape remains unclear. Some authors argue for a difference in susceptibility between the virus in the blood and CSF with selection of resistant mutant virus in the CNS (3) while others suggest a compartmentalization of the virus in the CNS (12). HIV is commonly present in the CNS during primary viremia presumably carried by CD4+ T Cells and monocytes. As infection progresses, the virus infects parenchymal macrophages and microglial cells with local replication and therefore generates the possibility of escape under poor CNS penetration regimen (13). CSF HIV RNA escape's clinical expression is likely related to neuroinflammation caused by the virus in the CNS in subjects with relatively preserved immune function. This inflammatory hypothesis is reinforced by the presence of surrogate markers of CNS inflammation (CSF pleiocytosis and increased proteinorachia, and white matter hyperintensities on T2-weighted and FLAIR imaging) as found in our patient (2, 9).

Risk factor for CSF escape are low nadir of CD4, low CD4/CD8 ratio and Protease Inhibitors (PI)-based regimens, all of which were present in our patient (13). An ART regimen with a good penetration in the CNS diminishes the symptomatology of HIV CSF escape (14). In our case the reintroduction of dolutegravir improved the control of virus CNS infection as demonstrated on following CSF samples at 2 and 4 months (Figure 2) with concomitant highly significant improvement in her clinical evolution. The effectiveness of ART on the CNS can be assessed using the CNS penetration effectiveness (CPE) method. This score is based on the effectiveness of the drugs in the CNS (pharmacodynamics), drugs concentration in the CNS (pharmacokinetics) and drugs characteristics (e.g., Protein bindings). The value for all drugs in the regimen are then summed to obtain a CPE value for the regimen (14, 15). In our patient's case, it is noteworthy that the CPE of the regimen before the change was 7 (Dolutegravir 4, Darunavir/Cobicistat 3) and 8 thereafter (Abacavir 3, Lamivudine 2, Darunavir/Ritonavir 3). However, Dolutegravir has the highest CPE rank and is a highly potent drugs which may explain the CSF escape when it has been stopped.

Mild parainfectious as well as HIV-related OMAS could have a spontaneous resolution whereas persistant ones require immunotherapy (4, 8). Review of previous cases of OMAS related to HIV shows that that treatments administered are heterogeneous. In most cases, the outcome is favorable regardless of the chosen therapeutic approach. While immunosuppressive therapy was not systematically used, HIV infection control was systematically applied (Supplementary Files). Therefore, the additive role to ART's shift on OMAS of intravenous pulsed corticotherapy and gabapentin in the present case remains hypothetical. Interestingly, these two medications were not described by Wang et al. Our patient showed a dramatic improvement not only of motor symptoms but also of behavioral disturbances.

HIV infection, related CNS inflammation due to HIV CSF escape as well as sinus thrombosis are associated with epilepsy, mainly when complicated by CNS structural lesions of opportunistic infection (1, 16). However, whether patients really presented with epilepsy on admission was unclear. Review of electroencephalographic patterns, presenting non-rythmic fronto-central activity could be attributed to multi-directional chaotic eyes movement and initial clinical picture could have been due to OMAS alone. Chronic HIV infection increases the risk of developing a deep veinous thrombosis even when infection is controlled. Pro-inflammatory state, decreased anticoagulant mechanisms, increased procoagulant factor, related immunodeficiency state and protease inhibitor may contribute to thrombotic events in HIV patients (16, 17). Noticeably, sinus thrombosis in HIV patients is paucisymptomatic and affects mostly superior sagittal and lateral sinus thrombosis (17). We decided to initiate Dabigatran on the basis of recent randomized study (18).

## Conclusion

We report a case of CSF HIV RNA escape complicated by OMAS and poorly symptomatic sinus thrombosis. Addition of dolutegravir to her HIV medication, a 5 days pulsed corticotherapy and anticoagulant treatment were associated with an excellent clinical evolution, restoring complete autonomy, and undetectable viral load on CSF and plasma at 4 months.

This case report highlights the necessity to evaluate HIV viral load on CSF facing neurological symptoms and reinforces the need to screen for a possible CSF virus escape. We suggest that CSF HIV escape is a new cause of OMAS. Proper virus control under specific ART may participate to good clinical outcome in such condition.

## Data Availability Statement

The original contributions presented in the study are included in the article/Supplementary Material, further inquiries can be directed to the corresponding author/s.

## Ethics Statement

Written informed consent was obtained from the individual(s), and minor(s)' legal guardian/next of kin, for the publication of any potentially identifiable images or data included in this article.

## Author Contributions

All authors listed have made a substantial, direct and intellectual contribution to the work, and approved it for publication.

## Conflict of Interest

The authors declare that the research was conducted in the absence of any commercial or financial relationships that could be construed as a potential conflict of interest.
