# Corrigendum: CSF HIV RNA Escape in Opsoclonus-Myoclonus-Ataxia Syndrome: Case Report and Review of the Literature

**DOI:** 10.3389/fneur.2021.665996

**Published:** 2021-03-17

**Authors:** Pierre Cabaraux, Arthur Poncelet, Jérome Honnorat, Remy Demeester, Soraya Cherifi, Mario Manto

**Affiliations:** ^1^Unité des Ataxies Cérébelleuses, Service de Neurologie, Centre Hospitalier Universitaire (CHU)-Charleroi, Charleroi, Belgium; ^2^Service de Médecine Interne, Centre Hospitalier Universitaire (CHU)-Charleroi, Charleroi, Belgium; ^3^Centre National de Référence pour les Syndromes Neurologiques Paranéoplasiques, Hospices Civils de Lyon, Synatac Team, NeuroMyoGene Institute, INSERM U1217/CNRS UMR5310, University Claude Bernard Lyon 1, Université de Lyon, Lyon, France

**Keywords:** HIV, CSF escape, opsoclonus myoclonus ataxia syndrome, cerebellum, sinus thrombosis

In the original article, the authors' names were spelled incorrectly. The forenames and surnames were interchanged. The corrected author list is shown below:

Pierre Cabaraux^1^^*^^†^, Arthur Poncelet^2^^†^, Jérome Honnorat^3^, Remy Demeester^2^, Soraya Cherifi^2^ and Mario Manto^1^

The authors' names have also been updated in the correspondence section, citation, article header, copyright statement, and any other relevant parts of the text.

The authors apologize for this error and state that this does not change the scientific conclusions of the article in any way. The original article has been updated.

